# Synthesis of FeSi–FeAl Composites from Separately Prepared FeSi and FeAl Alloys and Their Structure and Properties

**DOI:** 10.3390/ma16247685

**Published:** 2023-12-17

**Authors:** Pavel Novák, Jiří Duda, Filip Průša, Kateřina Skotnicová, Ivo Szurman, Bedřich Smetana

**Affiliations:** 1 Department of Metals and Corrosion Engineering, University of Chemistry and Technology, Prague, Technická 5, 166 28 Prague, Czech Republic; dudaj@vscht.cz (J.D.); prusaf@vscht.cz (F.P.); 2 Department of Materials and Technologies for Vehicles, Faculty of Materials Science and Technology, VSB—Technical University of Ostrava, 17. listopadu 15, 708 00 Ostrava, Czech Republic; katerina.skotnicova@vsb.cz (K.S.); ivo.szurman@vsb.cz (I.S.); 3 Department of Chemistry and Physico-Chemical Processes, Faculty of Materials Science and Technology, VSB—Technical University of Ostrava, 17. listopadu 15, 708 00 Ostrava, Czech Republic; bedrich.smetana@vsb.cz

**Keywords:** composite, iron silicide, iron aluminide, separate synthesis, mechanical properties

## Abstract

Composites consisting of iron aluminide and iron silicide phases were studied in this work. Powders of iron aluminide and iron silicide were prepared by mechanical alloying separately. Subsequently, they were blended in three different proportions and sintered by the SPS method under various conditions. After sintering, the composites are composed of FeAl and amounts of other silicides (Fe_5_Si_3_ and Fe_3_Si). Ternary Fe–Al–Si phases were not determined, even though their presence was predicted by DFT calculations. This disagreement was explained by steric factors, i.e., by differences in the space lattice of the present phases. Hardness and tribological properties were measured on composites with various weight ratios of iron aluminide and iron silicide. The results show that sintered silicides with the matrix composed of iron aluminide reach comparable hardness to tool steels. The composites with higher mass ratios of iron aluminide than silicide have higher hardness and better tribological properties.

## 1. Introduction

Cemented carbides, usually WC–Co cermets, are nowadays among the most widely used tool materials, together with the tool steels. The cemented carbides excel in the combination of wear resistance, strength, toughness and thermal stability. However, the sudden utilization of these materials could become problematic in the European Union, because the raw materials are not readily available in Europe. Therefore, the European Commission have listed cobalt and tungsten, i.e., the main components of cemented carbides, as critical raw materials (CRMs) since 2011 [1]. Critical raw materials have high economic importance together with high supply risk. The definition of this list launched the initiatives leading to the improved recycling of these raw materials and their potential substitution. In the case of WC–Co, the considered substitution could be of three approaches: substitution of tungsten carbide by another hard phase, substitution of cobalt binder by alternative metal or alloy, or total substitution of the whole material [2]. In the third approach, there were proposed solutions based on the TiC–Ni (or nickel alloy) system [3,4,5] or other metal–ceramics composites [6]. However, none of them are able to offer a similar combination of properties as WC–Co.

In our recent research, we figured out that the potential substitution could be based on hard intermetallics. For example, the Ti–Al–Si alloys were found to be highly wear resistant. These alloys, which are in fact TiAl–Ti_5_Si_3_ in situ composites, were found to be highly wear resistant thanks to the very hard Ti_5_Si_3_ silicide phase [7]. On the other hand, their toughness was found to be insufficient and even lower than corundum ceramics, for example [8]. When the same preparation route—mechanical alloying of the mixture of elemental powders—was used for the preparation of desired FeSi–FeAl composites, the result was completely different. There were FeAl, FeSi, but also a significant amount of Fe–Al–Si ternary phases [9]. And these ternary phases lead to high brittleness of this alloy. 

In the iron–silicon system, there are three silicides stable at room temperature (Fe_3_Si, FeSi and FeSi_2_) and one with a range of stability starting at 825 °C (Fe_5_Si_3_) [10]. Iron silicides are found in nature in some minerals like naquite (FeSi) [11] and gupeiite (Fe_3_Si), mostly through their meteoritic origin [12]. Rather than the hard phases, iron silicides are known for their semiconducting (FeSi and FeSi_2_) [13,14] and magnetic properties. Fe_3_Si and FeSi are ferromagnetics with soft magnetic behaviour [15,16]. Therefore, there are not many data about the mechanical properties of silicides. The data which are available, indicate that the FeSi phase is the hardest silicide stable at room temperature, and has the indentation hardness of 9.3 GPa [17] which is theoretically calculated as 10.95 GPa [18].

Stable iron aluminides are of four compositions—Fe_3_Al, FeAl, Fe_2_Al_5_, FeAl_2_ and FeAl_3_ (also denoted as Al_14_Fe_3_) [19], and except for the Fe_3_Al and FeAl phase, they are extremely brittle [20]. The latter mentioned phases (Fe_3_Al and FeAl) are considered as oxidation resistant and thermally stable and therefore they were already applied as the basis of refractory materials, such as Pyroferal [21]. These iron aluminides are generally softer than the above-described silicides, reaching the hardness of 220–450 HV [22].

In order to reach good mechanical properties, modern powder metallurgy methods are applied more commonly. Mechanical alloying is a method which uses high energy milling in order to transform the elemental powders to alloys or compounds [23]. During the process, repeated welding, fracturing, and rewelding of powder particles proceeds in the high energy ball mill, together with the severe plastic deformation of the powders. Therefore, the resulting materials have an ultrafine grained structure and improved mechanical properties [24]. Usually, mechanical alloying is considered as a long-term process, but there were several successful attempts to optimize the processing parameters to shorten its duration to tens of minutes or several hours [25]. To preserve the ultrafine grained structure reached by mechanical alloying and corresponding mechanical properties, advanced consolidation techniques, such as spark plasma sintering, should be applied. Spark plasma sintering is a method which uses an electric current to supply the heat for sintering, together with simultaneous application of pressure [26]. The role of electric current in the spark plasma sintering of metals is especially important in the heating of the sintered material by Joule heat; this is caused by electric resistivity of the material, possibly supported by the connected self-propagating reactions, e.g., with the formation of intermetallics and also a possible formation of the electric discharge between the sintered particles [26], while the pressure helps to minimize the porosity. The latter mentioned role of the electrical current is still controversial, since there exists both studies confirming it [27] and also opposing it [28]. The main indication, which supports the possible occurrence of discharge leading to plasma, is the fact that the surface of particles have a different structure after sintering than the core. It shows that there was significantly higher temperature on the surface of particles than in their cores [29]. Regardless of the mechanism, which is still not fully understood, the main advantages of the process are lower temperatures required for sintering and shorter sintering times (usually in minutes), which minimizes grain coarsening and saves energy [30].

From the above-mentioned text, it could be concluded that the combination of FeSi as the hard phase and iron aluminide as the matrix could be good for use as a machining tool material, because the aluminide phase can potentially provide the required toughness and oxidation resistance when the tool is heated due to the machining process, while the iron silicide can provide the hardness and wear resistance. Therefore, this work aims to focus on the synthesis and characterization of FeSi–FeAl composites by the separate synthesis of FeAl and FeSi phases and their blending and sintering. Since the coexistence of FeAl and FeSi phases is not thermodynamically stable without the ternary Fe–Al–Si phase, it is needed to determine the sintering conditions, which will lead to none or at least a minimized reaction between these phases.

## 2. Materials and Methods

The FeSi–FeAl composites were prepared by separate synthesis of iron aluminide and iron silicide by mechanical alloying. The mixtures of the chemical composition corresponding to FeAl and FeSi were prepared from elemental pure powders, which were mixed in appropriate amounts forming 20 g powder batches for mechanical alloying. The following powders were used to prepare the blend for mechanical alloying: Fe (purity 99.9%, particle size < 44 μm, Strem Chemicals, Newburyport, MA, USA), Al (purity 99.7%, particle size < 44 μm, Strem Chemicals) and Si (purity 99.5%, particle size < 44 μm, Alfa Aesar, Haverhill, MA, USA). Mechanical alloying of the blends was carried out in a planetary ball mill PM100 (Retsch, Haan, Germany), where the milling jar and also the milling balls were made of AISI 420 stainless steel. Argon (purity 99.996%) was used as the protective atmosphere during mechanical alloying. The mechanical alloying conditions were the following: duration of 10 h, change of rotation direction every 15 min, rotational velocity of 400 rpm, batch of 20 g and the ball-to-powder weight ratio of approx. 15:1.

In order to estimate the suitable sintering conditions, a mixture of 1 g of silicide and 1 g of aluminide was homogenized mechanically. Consequently, differential thermal analysis (DTA) using a Setaram Setsys 18TM device (Kep Technologies, Lyon, France) with a heating rate of 10 °C/min in an argon atmosphere (flow 80 mL/min) with a purity of 99.9999% was applied. The sample (31 mg) was placed in a corundum crucible during the analysis.

The FeSi and FeAl powders were mechanically blended in weight ratios of 1:2, 1:1 and 2:1. The FeSi–FeAl blends of mechanically alloyed powders were consolidated to cylindrical samples with a 20 mm diameter by spark plasma sintering (SPS, FCT Systeme HP-D10, Rauenstein, Germany) at the temperatures of 850 and 1000 °C with a pressure of 70 MPa for 15 min. The heating rate of 300 °C/min was used. Controlled cooling with the rate of 50 °C/min was applied after the sintering process to prevent excessive stress accumulation in the materials. After sintering at 850 °C, additional processing by hot isostatic pressing (HIP) was tested for reduction of porosity. The HIP process was carried out at a temperature of 850 °C under a pressure of 150 MPa for a duration of 1.5 h and a heating rate of 10 °C/min.

The prepared composites were characterized from the viewpoints of phase composition, microstructure, and mechanical and tribological properties. The phase composition of the mechanically alloyed powders and consolidated samples were examined by X-ray diffraction (XRD) analysis by the means of a X’Pert Pro diffractometer (PANalytical, Almelo, The Netherlands) using CoK radiation.

The microstructure of the materials in the state of mechanically alloyed powders and consolidated samples was observed using a VEGA 3 LMU (TESCAN, Brno, Czech Republic) scanning electron microscope equipped with an energy-dispersive spectrometer X-max 20 mm^2^ (EDS, Oxford Instruments, High Wycombe, UK), which was also used for local analysis of elemental composition. 

In addition to the phase analysis and microstructure evaluation, the samples were characterized from the viewpoints of mechanical properties (hardness), as well as tribological behaviour (wear rate and friction coefficient). Hardness was measured by the Vickers method at a load of 10 kg (HV 10) which corresponds to 9.8 N. The hardness was measured ten times on each sample and the average values were calculated. The wear resistance was measured on a TriboTester tribometer (Tribotechnic, Clichy, France) using the ball-on-disc tribometer in the linear reciprocal mode (*e* excenter of 5 mm). In this case, the “ball” was made of alumina (α-Al_2_O_3_), and it had 6 mm in diameter and the “disc” was a sample. No lubricant was used in the process. The sliding distance (*l*) in this process was 20 m and a normal force (*F*) of 5 N was used. The wear rate (*w* [mm^3^ N^−1^ m^−1^]) was calculated from the equation below, where we take into consideration the wear track section area (*A* [mm^2^]).

$$w=\frac{A\cdot e}{F\cdot l}$$

## 3. Results

### 3.1. Phase Composition of Mechanically Alloyed Powders

**Figure 1:**
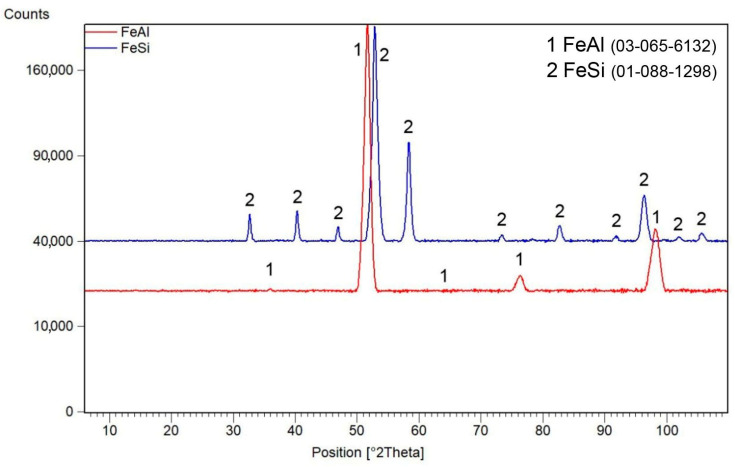
X-ray diffraction patterns of mechanically alloyed FeSi and FeAl powders. ICDD reference numbers are given in the legend.

After mechanical alloying, an X-ray diffraction analysis was performed in order to verify the composition of the powder materials, see Figure 1. Iron silicide FeSi (cubic, P213 space group, 4.4950 × 10^−10^ m) was formed by mechanical alloying of the Fe–Si powder mixture after milling for 10 h. In the case of iron and aluminium powders, iron aluminide FeAl (cubic, Pm-3m space group, a = 2.9076 × 10^−10^ m) was formed. No other phases were detected, so the powders can be considered as almost pure FeSi and FeAl phases, considering the detection limit of XRD. 

### 3.2. Thermal Analysis of FeSi–FeAl Mixture

**Figure 2:**
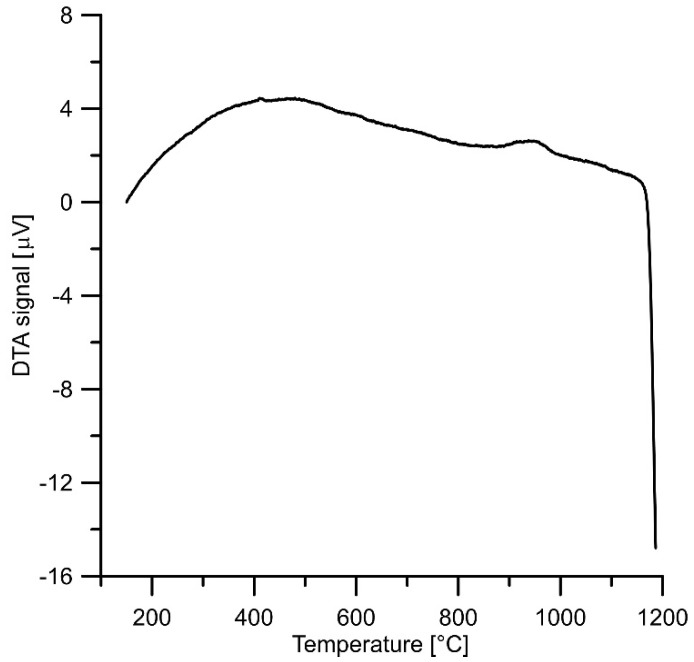
DTA heating curve of the FeSi–FeAl powder mixture.

The DTA results are shown in Figure 2. It can be seen from the DTA curve obtained during the heating process for the mixture of FeSi and FeAl, that some exothermic effect takes place between 870 and 1003 °C (accompanied by a relatively small amount of heat). Based on the previous results of the Fe–Al–Si system [31], it can be expected that this event corresponds to the reaction associated with the formation of undesirable Fe–Al–Si ternary phases. A strongly endothermic effect can be observed with the onset temperature of approx. 1150 °C, associated with melting which indicates reaching the solidus or eutectic temperature. The sintering temperature should be selected below the liquidus temperature in order to prevent melting. In addition, it is desirable to avoid any additional reaction in the system. Based on the results, two sintering temperatures were selected—the temperature of 850 °C, which is below any observed phase transformations and 1000 °C to describe what happens in this temperature interval. The sintering temperature of 1000 °C was previously used for sintering of Fe–Al–Si alloys [9].

### 3.3. Microstructure and Phase Composition vs. Sintering Conditions

**Figure 3:**
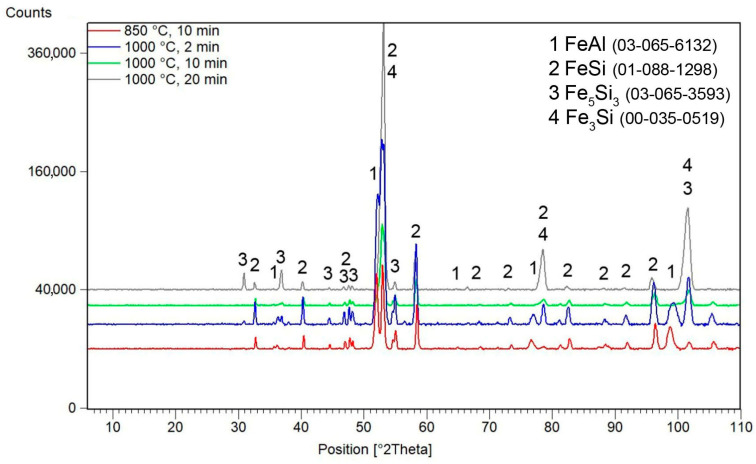
X-ray diffraction patterns of FeSi–FeAl composites sintered under various conditions. ICDD reference numbers are given in the legend.

The XRD patterns in Figure 3 show the dependence of the phase composition of FeSi–FeAl composite in a 1:1 weight ratio on the conditions of sintering by the SPS method. X-ray diffraction did not reveal the formation of any Fe–Al–Si ternary phases for any sample. Of the aluminide phases, the samples contain only the FeAl phase. In the sample sintered at 850 °C for 10 min, the FeSi (cubic, P213 space group, 4.4950 × 10^−10^ m) phase is the dominant silicide phase. In addition to this phase, there is also a minor amount of the Fe_5_Si_3_ (hexagonal, P63/mcm space group, a = 6.7552 × 10^−10^ m, c = 4.7174 × 10^−10^ m).

The samples sintered at 1000 °C contain, in addition to the FeAl phase, the following iron silicides: FeSi, Fe_5_Si_3_ and Fe_3_Si (cubic, Im-3m, a = 2.8410 × 10^−10^ m). At this temperature and pressure, as the holding time increases, the representation of Fe_3_Si silicide increases at the expense of the FeSi and Fe_5_Si_3_ silicides, as seen in Figure 3.

**Figure 4:**
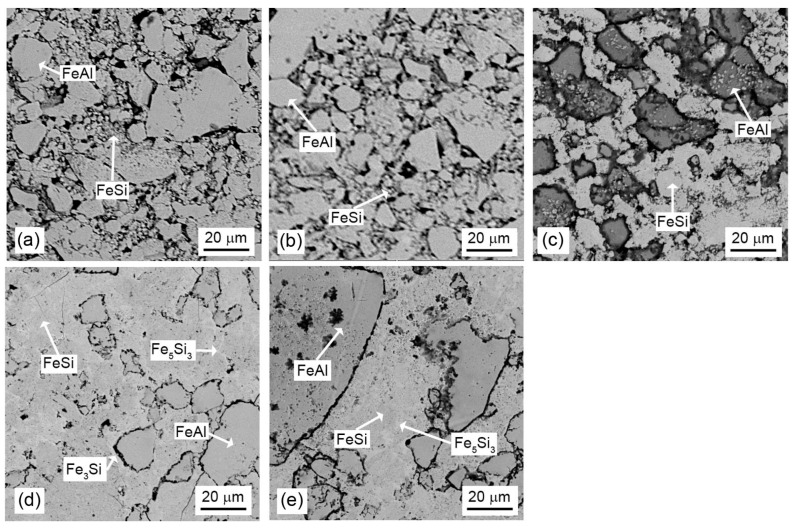
SEM micrographs of FeSi–FeAl composites with a silicide-to-aluminide weight ratio of 1:1 sintered under various conditions: (**a**) 850 °C, 10 min, (**b**) 850 °C, 10 min and processed by HIP, (**c**) 1000 °C, 2 min, (**d**) 1000 °C, 10 min and (**e**) 1000 °C, 20 min.

The microstructure of the composites vs. the sintering conditions is presented in Figure 4. The SEM micrograph of the sample sintered at 850 °C for 10 min (Figure 4a) indicates that this sample was not fully sintered by the SPS method at 850 °C. It can be seen from the micrograph that it contains numerous pores. Image analysis with the use of the ImageJ program revealed that the porosity of this sample is approx. 40 vol. %. The sample itself disintegrated into smaller pieces after being removed from the graphite die after sintering. Its hardness was 164 HV 10 due to the insufficient consolidation of the sample. In order to reduce the porosity, another sample sintered by the SPS method at 850 °C was pressed by HIP technology. The same temperature used for sintering (850 °C) was used for HIP in order to avoid any transformation, based on the DTA results (Figure 2). The general role of HIP is the healing of internal porosity by the simultaneous action of pressure and temperature. The sample that underwent HIP showed a lower porosity (Figure 4b), but it was still high (around 30 vol. %) compared to the samples sintered at 1000 °C (Figure 4c–e). A sintering temperature of 850 °C is therefore unusable for sufficient sintering of the material in any way.

The materials sintered at 1000 °C contain a smaller amount of pores (less than 5% by volume). Therefore, a temperature of 1000 °C is more suitable for sintering using the SPS method, and for a mass ratio of silicide to aluminide of 1:1, it is sufficient for sintering the sample without the formation of ternary phases.

### 3.4. Microstructure and Phase Composition vs. FeSi:FeAl Ratio

**Figure 5:**
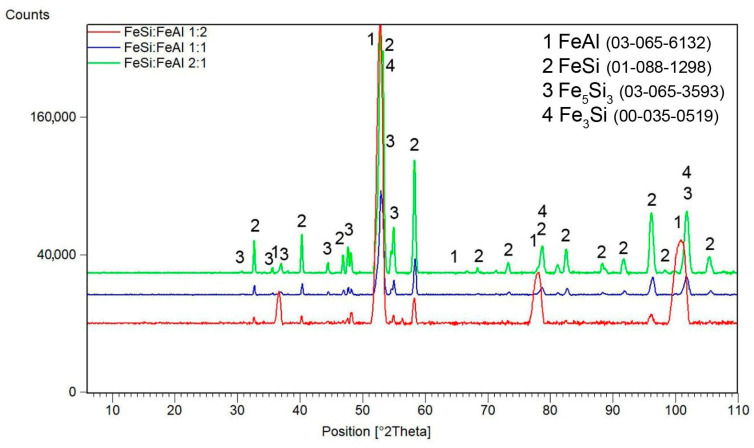
X-ray diffraction patterns of FeSi–FeAl composites with a variable FeSi:FeAl ratio. ICDD reference numbers are given in the legend.

From the above-mentioned results, it can be concluded that the temperature of 850 °C is absolutely insufficient for the sintering of these composites, while sintering at 1000 °C seems to be feasible. For this reason, the sintering at 1000 °C for 10 min was applied to investigate the effect of the silicide-to-aluminide ratio on the phase composition and microstructure. The phase composition of these composites is summarized in Figure 5. The XRD analysis did not reveal the formation of any unwanted ternary phase. All of the samples contain FeAl as the only detectable aluminide phase. The FeSi:FeAl 1:1 sample contains silicide phases of FeSi and Fe_5_Si_3_, with a small amount of Fe_3_Si as well. The FeSi:FeAl 2:1 sample is rich in silicide phases due to the higher content of iron silicide in the initial powder mixture. It also contains FeSi, Fe_5_Si_3_ and Fe_3_Si phases. In the FeSi:FeAl 1:2 sample, the FeAl phase predominates due to the higher content of iron aluminide in the starting mixture. Among the silicide phases, it also contains Fe_5_Si_3_ silicide in smaller amounts, in addition to FeSi silicide. The representation of the Fe_3_Si phase is minimal here.

**Figure 6:**
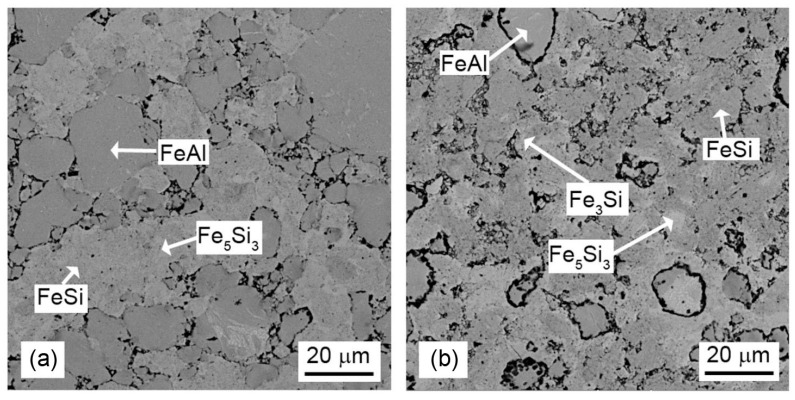
SEM micrographs of FeSi–FeAl composites with various silicide-to-aluminide weight ratios: (**a**) 1:2, (**b**) 2:1.

The microstructure of the composites varies with the changing ratio between the silicide and aluminide phase. In the case of the FeSi:FeAl ratio of 1:2, there is a predominance of iron aluminide. On the other hand, for the opposite ratio, the silicide is the dominant phase (see Figure 6). The average chemical composition of the aluminide and silicide phases is presented in Table 1. It can be seen that both silicon in silicide and aluminium in aluminide are partially substituted by aluminide and silicide, respectively. The contamination by chromium was caused by the wear of the stainless-steel balls during milling. 

### 3.5. Mechanical and Tribological Properties

The hardness of a series of samples sintered at 1000 °C with a sintering time of 10 min is shown in Table 1. Although the FeSi:FeAl 2:1 sample contains a harder silicide phase than the aluminide binder, it reaches a lower hardness value than the FeSi:FeAl 1:2 sample. Moreover, it is evident that changing the mass ratio of aluminide and silicide content from 1:1 leads to an increase in the hardness of the material.

Two wear tracks were realized on each sample by the test against the standardized alumina ball. The values shown in Table 1 are arithmetic averages of measurement results from individual tracks. Table 1 shows the friction coefficient values for a series of samples. It can be seen that changing the ratio of aluminide to silicide from 1:1 to either side leads to a reduction in the coefficient of friction.

In the case of the wear rate, the values are decreasing with an increasing amount of the aluminide phase in the composite (Table 2). The lowest wear rate, similar to the value of the coefficient of friction, is shown by the sample FeSi:FeAl 1:2.

**Figure 7:**
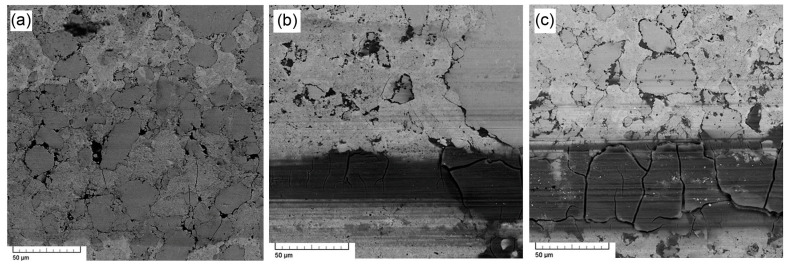
SEM-BSE micrographs of the wear tracks on the sample (**a**) FeSi:FeAl 1:2, (**b**) FeSi:FeAl 1:1, and (**c**) FeSi:FeAl 2:1.

Images (SEM) of individual wear tracks are shown in Figure 7. Measurements on the FeSi:FeAl 1:1 and 2:1 samples caused numerous visible cracks. As a result of friction, there was also obvious oxidation in the wear track on these samples, visible as dark areas in the BSE mode on the samples.

For the FeSi:FeAl 1:2 sample, oxidation of the sample and abrasive wear also occurred, but not to such an extent. The reason being the larger ratio of the binder aluminide phase, which makes it more difficult to pull out the particles and which is also makes it more resistant to oxidation.

## 4. Discussion

**Figure 8:**
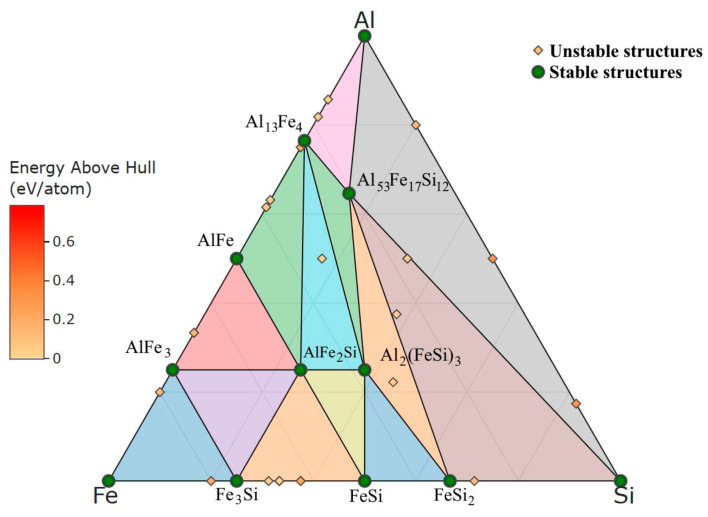
Phase diagram of stable and unstable Al–Fe–Si structures obtained by DFT calculations (at.%) at a theoretical temperature of 0 K.

In this work, the new concept of the preparation of Fe–Al–Si-based materials was tested. As stated in the introduction, our previous research focused on these materials led to alloys with the presence of a high-volume fraction of unwanted brittle Fe–Al–Si ternary phases [9]; as was expected on the basis of the Fe–Al–Si equilibrium ternary phase diagram [32], when the manufacturing route started from blended iron, aluminium and silicon powders. Based on our own DFT (density functional theory) calculations, a phase diagram of all possible stable structures for the Al–Fe–Si system was obtained. Al_13_Fe_4_, AlFe, AlFe_3_, Fe_3_Si, FeSi and FeSi_2_ binary compounds and AlFe_2_Si, Al_2_(FeSi)_3_ and Al_53_Fe_17_Si_12_ ternary compounds are found to be stable—see Figure 8. In this work, we tried the typical method for the manufacturing of composites, i.e., separate preparation of matrix (iron aluminide) and particulate reinforcement (iron silicide) by mechanical alloying. In this case, the occurrence of the Fe–Al–Si ternary phases was not observed for any sintering conditions. Why do these phases not form, even though they are thermodynamically stable under the sintering conditions? The reasons are most likely due to the kinetics of their formation, the mutual diffusivities of iron, aluminium and silicon, and also the differences in the crystal structure of the phases. In the case of the latter point, there is a blend of iron aluminide (cubic, Pm-3m space group, a = 2.9076 × 10^−10^ m) and iron silicide (cubic, P213 space group, 4.4950 × 10^−10^ m) before sintering. Both phases have a cubic structure, even though there is a relatively large misfit in lattice parameters. However, the expected Fe_2_Al_3_Si_3_ phase, which was previously detected in Fe–Al–Si materials prepared from pure elements directly, differs strongly in crystal structure from the initial phases (monoclinic, P21/n space group, a = 7.1790 × 10^−10^ m, b = 8.3540 × 10^−10^ m, c = 14.4550 × 10^−10^ m, α = γ = 90°, β = 93.8°) [33]. It can be expected that the steric effect can block the formation of this phase in the solid state during sintering, when there are already existing phases with a more similar structure. The formation of the Fe_2_Al_3_Si_3_ phase with much larger lattice parameters than the original FeAl and FeSi phases is clearly not preferred. Instead of the ternary phases, other types of iron silicides appeared. There was Fe_5_Si_3_ (hexagonal, P63/mcm space group, a = 6.7552 × 10^−10^ m, c = 4.7174 × 10^−10^ m) and Fe_3_Si (cubic, Im-3m, a = 2.8410 × 10^−10^ m) present after sintering, while the amount of the Fe_3_Si phase was increasing with the sintering temperature and time. Based on the above considerations regarding the structure of the phases, these results are logical because the Fe_3_Si phase has a structure very similar to FeAl. In the case of the Fe_5_Si_3_ phase, the explanation of its presence is not so intuitive and clear. This phase is not thermodynamically stable, and its region of stability starts at 825 °C. The reasons for its stabilization could be in the sudden changes and local differences of temperature during the spark plasma sintering, when we consider the presence of discharges between the particles [27,34]. In addition, the steric effect can play a role again. This phase, despite being hexagonal, has the “c” lattice parameter very close to the FeSi phase and an “a” almost double that of the lattice parameter of FeAl.

In addition to the proved feasibility of the preparation of these FeSi–FeAl composites, another important aspect of this work is the determination of their wear resistance. This parameter is crucial when the material is considered a future tool material. It was determined that the resistance to dry sliding wear against the alumina ball increases with an increasing amount of the aluminide phase, even though this phase was expected to have lower hardness based on the published data about the hardness of FeSi [17] and FeAl [22]. The first possible reason for this behaviour is that the aluminide phase being the one with higher ductility and toughness, prevents the silicide particles from pulling out from the composite during the wear test. This mechanism was also confirmed in published cases with different materials [35]. The negligible release of silicide particles also confirms good bonding between the silicide and aluminide in the composites. The observation of the wear track, however, also identified another effect. The wear track was highly oxidized in the case of the composites with lower amounts of aluminide. Because the friction coefficients are rather high (see Table 2), the large fraction of normal force is transferred to the friction force, thus causing the local heating of the material. And our previous results confirmed that iron aluminide is slightly more resistant to high-temperature oxidation than iron silicide [36]. The high degree of oxidation in the wear track causes the release of oxidized wear debris and promotes abrasive wear because of the hard nature of the aluminium and silicon oxides. The determined wear rates are comparable to conventional hot-work tool steels [37] and slightly lower than high grade cold-work tool steels [38], while the hardness almost reaches the appropriate level of heat-treated cold-work tool steels [38].

The use of sintered silicides as a new material for the production of machining tools seems to be promising. It may be possible to improve the mechanical and tribological properties, when the optimal ratio of aluminide and iron silicide in the starting mixture is optimized, especially in the area with greater representation of aluminide than silicide. The important benefit of these materials is that the above-presented level of wear resistance can be reached without any heat treatment in the case of the investigated FeSi–FeAl composites.

## 5. Conclusions

In this work, composites formed by the iron aluminide binder phase and the iron silicide as the particulate reinforcement were investigated. Using thermal analysis of a mixture of aluminide and iron silicide in a 1:1 ratio, slightly exothermic reactions were found to occur between 850 °C and 1000 °C between the phases present. The work therefore examined samples sintered by the SPS method at temperatures of 850 °C and 1000 °C. The material sintered at 850 °C by the SPS method showed high porosity. In order to reduce porosity, another sample sintered by the SPS method at 850 °C was pressed by HIP technology. The HIP process led to a slightly lower porosity, but it was still high compared to the samples sintered at 1000 °C. A sintering temperature of 850 °C is unusable for sufficient densification of the material. A temperature of 1000 °C is suitable for sintering samples using the SPS method. The porosity of individual samples sintered at this temperature was low, even at short times of sintering. Similar to the sintered samples at 850 °C, no undesirable ternary phases and a single aluminide phase of FeAl were formed during sintering. As the holding time at a temperature of 1000 °C increases, the amount of the Fe_3_Si silicide phase increases at the expense of the FeSi and Fe_5_Si_3_ phases. The microstructure of the samples sintered at 1000 °C was fine-grained in all cases. During the course of this work, the influence of the mass ratio of aluminide and iron silicide was also investigated, as well as the starting mixture’s phase composition and mechanical properties. It showed that samples with a higher aluminide amount (FeSi:FeAl 2:1) had the highest hardness value. The hardness and wear resistance are almost comparable to conventional tool steels, but without the need of any heat treatment. Therefore, these materials can be potentially considered as a green alternative to tool steel, because of the lower carbon footprint associated with their processing.

## Figures and Tables

**Table 1:** Average chemical composition of the silicide and aluminide phase (EDS, in at.%).

Phase	Fe	Si	Al	Cr
FeAl	47.5 ± 3.0	12.9 ± 2.8	37.5 ± 3.0	2.2 ± 0.2
FeSi	46.5 ± 0.4	41.0 ± 0.4	11.3 ± 0.3	1.4 ± 0.3
Fe_5_Si_3_	60.1 ± 1.8	24.4 ± 3.6	13.4 ± 4.0	2.2 ± 0.3

**Table 2:** Hardness (HV10) and tribological properties of tested composites against the Al_2_O_3_ ball, f—friction coefficient, w—wear rate.

FeSi:FeAl Ratio	HV10	f	*w* (mm^3^ N^−1^ m^−1^)
1:2	720 ± 38	0.466	$1.6\times 10^{-6}$
1:1	628 ± 33	0.539	$4.1\times 10^{-5}$
2:1	664 ± 33	0.473	$4.5\times 10^{-4}$

## Data Availability

The data presented in this study are available on request from the corresponding author.
